# Dataset of sentiment tagged language resources for Bosnian language

**DOI:** 10.1016/j.dib.2024.110247

**Published:** 2024-02-28

**Authors:** Sead Jahić, Jernej Vičič

**Affiliations:** a University of Primorska, FAMNIT, Glagoljaska 8, 6000 Koper, Slovenia; b Research Centre of the Slovenian Academy of Sciences and Arts, The Fran Ramovž Institute, Novi trg 2, 1000, Ljubljana, Slovenia

**Keywords:** Stopwords, Lexicon, AnAwords, Intensifiers, Diminishers, Sense

## Abstract

The Bosnian language holds significant importance as a member of the West-South Slavic subgroup within the Slavic branch of the Indo-European linguistic family. With approximately 2.5 million speakers in Europe, including 1.87 million individuals in Bosnia and Herzegovina alone, the Bosnian language constitutes the mother tongue for a considerable portion of the population.

In Natural Language Processing (NLP) tasks related to the Bosnian language, besides removing stop words, it is important to consider the influence of other linguistic elements. Bosnian text contains words derived from diminishers, relative intensifiers, minimizers, maximizers, boosters, and approximators. These words contribute to the overall meaning and sentiment analysis of the text. By including these elements in NLP models and algorithms, researchers can achieve more accurate and nuanced analysis of Bosnian language data, enhancing the effectiveness of NLP applications.

The two lists of sentiment annotated words that present the core of the Bosnian sentiment-annotated lexicon, a list of the stopwords, and a list of Affirmative and non-Affrimative words (AnAwords) composed mostly of intensifiers and diminishers, were used to construct a dataset that presents the base for sentiment analysis in the Bosnian language.

Specifications Table

**Table:** 

Subject	Computer Science Application
Specific subject area	Lexicon, AnAwords, Attenuators, and Stopwords Datasets
Data format	Analyzed, raw
Type of data	Table
Data collection	Several parameters were considered for data collection, including the size and diversity of the texts, the representativeness of the language used, and the specific requirements for sentiment analysis in the Bosnian language. For the lexicon, we utilized the KSS 1.1 Slovene opinion lexicon, which contains 5125 negative lemmas and 1911 positive lemmas which is described in sections 4 and 5 and subsection 5.1. As for the AnAwords, we used lists provided by Patra et al. [4], Pintarić et al. [5]., and by Osmankadić [6], more in subsection 5.2. For the stopwords, we employed the Croatian stopwords list provided by the SpaCy package in Python, which were extended and described in section 5 and subsection 5.3.
Data source location	Primary data source (partial): Slovenian sense annotated lexicon presented in Kadunc [17]. Data source location: University of Primorska, UPFAMNIT, Glagoljaska 8, 6000 Koper, Slovenia
Data accessibility	Sentiment polarity lexicon of Bosnian language Repository: Zenodo DOI: 10.5281/zenodo.10373141 URL: https://doi.org/10.5281/zenodo.10373141 AnAwords list, Stopwords list, and Polarity Shifters list Repository: Zenodo DOI: 10.5281/zenodo.10373107 URL: https://doi.org/10.5281/zenodo.10373107
Related research article	Jahić and Vičič [1] present an impact of negation and attenuators on the overall quality of sentiment tagging process for Bosnian language.

## 1 Value of the Data

• The presented dataset of sentiment analysis resources for Bosnian language in form of a lexicon, a list of Affirmative and non-Affrimative words (AnAwords), and a list of stopwords holds significant value for a range of applicable and research projects.
• These data are useful for research dealing with embedding dual graphs on surfaces, interdisciplinary research between topology and combinatorics. Furthermore, the dataset can be used in industry or art to design/create new surfaces.
• These are the identified potential users of the data: Researchers and developers working on sentiment analysis benefit from the reduced text size and the incorporation of AnAwords. Developers of automatic information retrieval systems find that eliminating stop words simplifies the retrieval process, improving both the quality and speed of the system, developers of online search engines and catalogs benefit from the removal of stop-words as it leads to more concise and faster searches.
• The data can be simply reused by other researchers by using the stopwords list as a lookup dictionary, allowing for easy detection and removal of stopwords from the analyzed texts. On the other hand, the AnAwords list plays a crucial role in identifying amplifiers that have a significant impact on nearby words, intensifying their meaning. The sentiment lexicon serves as a foundation for identifying sentiment-annotated words. By utilizing the lexicon, developers and researchers can effectively extract and differentiate words with sentimental value from the rest of the text, enhancing the overall analysis process.

## 2 Background

**Fig. 1:**
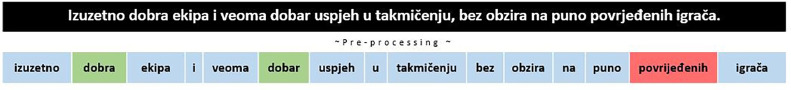
Detecting of the lexicon terms in example. Green highlighted presents positive-annotated term, while negative-annotated terms from lexicon are highlighted in a red color. (For interpretation of the references to color in this figure legend, the reader is referred to the web version of this article.)

**Fig. 2:**
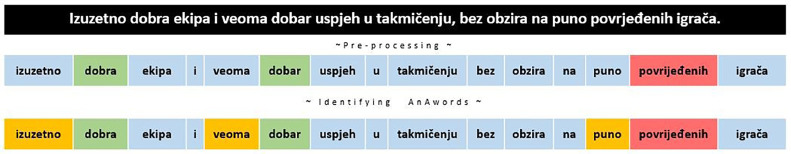
Detecting of the lexicon terms in example. Green highlighted presents positive-annotated term, while negative-annotated terms from lexicon are highlighted in a red color. (For interpretation of the references to color in this figure legend, the reader is referred to the web version of this article.)

**Fig. 3:**
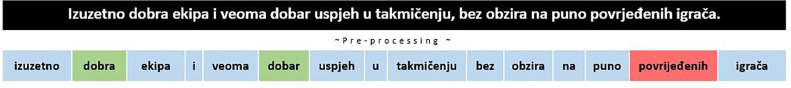
The AnAwords word stands out with an orange color, while the cells highlighted in green and red represent positively and negatively annotated words from the lexicon, respectively. (For interpretation of the references to color in this figure legend, the reader is referred to the web version of this article.)

The objective of this study is to present valuable resources for sentiment analysis in the Bosnian language, including a comprehensive sentiment lexicon, AnAwords list, and a stopwords list. These resources are essential for researchers and developers working on Natural Language Processing (NLP) tasks, as well as for applications such as online search engines and information retrieval systems. By providing these resources, we aim to facilitate sentiment analysis and improve the understanding of sentiment in Bosnian language texts (Fig. 1, Fig. 2, Fig. 3).

Most of the new research in NLP is based on Deep learning and statistical methods, but there is still a lot of need for hand-crafted language resources, particularly for under-resourced languages. Deep learning relies on big amounts of quality language data. The resources presented in this paper can also be used to enhance basic deep learning and statistical methods.

## 3 Data Description

Bosnian, as a language with its own unique characteristics, poses challenges in NLP tasks [2,3]. In the field of sentiment analysis, lexicon-based methods, also known as dictionary-based methods, are widely used to identify and classify the sentiment of the text. These methods rely on predefined lists of words or phrases, known as lexicons or dictionaries, which contain words associated with positive, negative, or neutral sentiment. Various lexicons are available for sentiment analysis, ranging from general-purpose lexicons to domain-specific lexicons. Some of the commonly used lexicons in sentiment analysis include SentiWordNet [7], AFINN [8], and the General Inquirer [9], which is one of the first-known human-annotated lexicons for sentiment analysis. These lexicons assign a semantic orientation or polarity to individual words, allowing sentiment analysis algorithms to calculate the overall sentiment of a text.

Many lexicon-based approaches have adopted these assumptions and have been applied to various languages, including Slavic languages. For example, the HowNet lexicon has been used for Chinese sentiment analysis [10], while researchers have developed lexicons specific to Slavic languages such as Bulgarian [11], Croatian [12], Czech [13], Macedonian [14], Polish [15], Slovak [16], and Slovenian [17].

The Bosnian sentiment lexicon comprises two files, one for positive words and the other for negative words. Basic information about the files is presented in Table 1, and the lexicon is available at Zenodo [18].

**Table 1:** The dataset of the sentiment-annotated lexicon of the Bosnian language: positive words and negative words.

File name	Number of entries
BOSNIAN NEGATIVE.txt	3935
BOSNIAN POSITIVE.txt	1219

A few examples from the aforementioned list (with English translation) are presented in Table 2.

**Table 2:** Examples of lexicon entries in original form and English translation, poditive followed by negative.

Lexicon entry	English translation
blažen	blessed
blaženstvo	bliss
blistav	dazzling
bodriti	(to) cheer
amputirati	(to) amputate
anarhija	anarchy

First goal of this article is the issue of automatic detection of sentiment-annotated words in the text written in the Bosnian language. For example, in the sentence “Izuzetno dobra ekipa i veoma dobar uspjeh u takmičenju, bez obzira na puno povrjeđenih igrača.” (Engl. “An extremely good team and a very good success in the competition, despite many injured players.”), words such as: ‘dobra’, ‘dobar’ (Engl. good), have positive meanings, and the word ‘povrijeđenih’ (Engl. injured) have a negative sentiment polarity.

The AnAwords list includes various linguistic elements such as intensifiers, diminishers, and other modifiers that influence sentiment analysis.

Stopwords are a collection of words that are intentionally filtered out or “stopped” during the natural language processing of textual data. These words usually consist of commonly used and frequently occurring terms in a language, which are deemed to contribute minimally or no significance in deciphering the meaning or context of a text.

Basic information about both files (AnAwords list, and stopwords list) is presented in Table 3, and the lexicon is available at Zenodo [19].

**Table 3:** The dataset of the sentiment-annotated lexicon of the Bosnian language: positive words and negative words.

File name	Number of entries
BOSNIAN_AnAwords_diminishers_2023.txt	49
BOSNIAN_AnAwords_intensifiers_2023.txt	89
BOSNIAN_polarity_shifters_2023.txt	34
BOSNIAN_stopwords_2023.txt	595

## 4 Experimental Design, Materials and Methods

### 4.1 The Bosnian sentiment-annotated lexicon

**Fig. 4:**
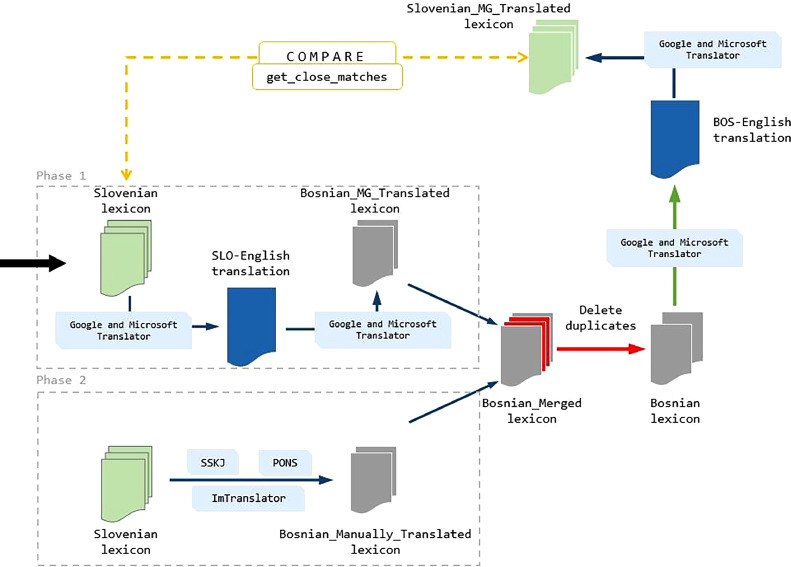
Construction and validation process of the Bosnian lexicon through two phases.

The process of creating the Bosnian sentiment-annotated lexicon involved two phases: translation and manual verification (see Fig. 4).

• In the initial phase the Slovene opinion lexicon KSS 1.1 [16] underwent translation into English through the utilization of Google and Microsoft translators. Subsequently, this intermediary English version was subjected to translation into the Bosnian language, which is visually depicted in Fig. 4. It is within this context that the ’Bosnian MG Translated’ lexicon was produced.
• The next phase was the creation of the lexicon in a two-phase manner. Firstly, words from the Slovenian lexicon were manually translated into Bosnian language. This comprehensive process encompassed a meticulous verification of each term using various tools, including Pons (https://sl.pons.com/), Google Translate, ImTranslator (https://imtranslator.net/), and the Dictionary of Slovenian Literary Language (SSKJ - Slovar slovenskega knjižnega jezika: https://fran.si/). The outcome of this phase yielded the ‘Bosnian Manually Translated’ lexicon. During our analysis, we discovered that among these multi-part words, some contained elements from the AnAwords list, which we treated separately. Examples of such cases include ‘hudo bolan’ (Bosnian: veoma bolan, English: very painful), ‘zelo poceni’ (Bosnian: veoma jeftin, English: very cheap), ‘povsem prava’ (Bosnian: potpuno pravo (tačno) English: completely right), and others. Additionally, we found that there were entire expressions in the Slovenian lexicon that were not translatable into the Bosnian lexicon (losing the original meaning). Some examples of such expressions are ‘nič hudega sluteč’ (Bosnian: ne slutiti ništa loše, English: unaware of any harm), ‘obesiti na klin’ (Bosnian: objesiti o klin, English: hang on a nail), ‘veliko hrupa za nič’ (Bosnian: mnogo buke oko ničega, English: much ado about nothing), ‘zvit kot lisica’ (Bosnian: lukav kao lisica, English: sly as a fox), and more.

Those two lexicons ‘Bosnian MG Translated’ lexicon and ‘Bosnian Manually Translated’ lexicon, were subsequently united and merged into a cohesive entity, referred to as the ‘Bosnian Merged’ lexicon. The refinement process further entailed the removal of duplicate words or lemmas. The final result was the initial iteration of the Bosnian sentiment lexicon. To ensure the utmost accuracy and robustness of the lexicon, a back-translation procedure was executed. This involved translating the newly composed Bosnian lexicon back into the Slovenian language, as depicted in Fig. 4.

The accuracy and quality of the developed resources were evaluated through back-translation and comparison with existing lexicons. The matching accuracy was calculated by comparing the translated lexicon with the original Slovenian lexicon. All translations were done by 2 human annotators (One Slovenian who speaks perfect Bosnian) and the other native Bosnian who speaks Slovenian very well. All translations were cross checked and differences discussed on a one-to-one basis.

## 5 The AnAwords List

The AnAwords list plays a crucial role in the detection of sentiment in textual data, particularly in the Bosnian language. This list was compiled based on existing research on intensifiers, diminishers, and other related linguistic elements. To ensure its relevance and accuracy for sentiment analysis in the Bosnian language, the list underwent a review and validation process.

In the context of intensifiers in the Bosnian, Croatian, Serbian, and Slovenian languages, there has been limited research on intensifiers. Pintarić and Frleta [5] provide insights into intensifiers, including maximizers, boosters, and moderators used in Croatian, with a focus on collocation. These intensifiers often collocate with adjectives, adverbs, and verbs across various semantic fields. The Croatian corpus consists of 33 intensifiers, including terms like “dozlaboga”, “krajnje”, “potpuno”, “vrlo”, and more.

The AnAwords list predominantly consists of adverbs of manner and adjectives, which are common intensifiers and diminishers in textual data. The creation of this list was inspired by Osmankadić [6], who identified six sublists of intensifiers: maximizers, boosters, approximators, relative intensifiers, diminishers, and minimizers. Another study by Patra et al. [4] also identifies six types of intensifiers, similar to Osmankadić´s findings, but classifies “relative intensifiers” as “compromisers” (in total 94 intensifiers).

The compilation and adoption of research from these various sources resulted in the comprehensive AnAwords list, specifically tailored for the Bosnian language. This involved translating and adapting terms to ensure their applicability in the Bosnian context. The final AnAwords list consists of 138 terms, offering valuable resources for sentiment analysis in the Bosnian language.

## 6 Stopwords

For the Bosnian language, we have created a comprehensive stopwords list by incorporating existing resources and applying manual curation. This list consists of common words, pro-nouns, prepositions, conjunctions, and other non-content words that are frequently encountered in Bosnian texts. The Bosnian stopwords list aids in improving the accuracy and efficiency of various NLP tasks, such as information retrieval and text classification.

The information about stopwords reveals that these words are commonly found in a language and are considered to have little or no significance in determining the meaning or context of a text. For our research, we initially utilized the stopwords list provided by the SpaCy package in Python for the Croatian language. We then translated these stopwords to fit the Bosnian language. However, we made certain additions to the Bosnian stopwords list that are not present in the Croatian list.

The Bosnian stopwords list includes all 30 letters specific to the Bosnian language, the names of all months (both in the Croatian and Bosnian pronunciations, such as “ožujak” and “mart”), as well as their abbreviations (e.g., “april” and “apr”). Additionally, we included Romanian numbers that can be represented by letters, such as ‘ix,’ ‘vii,’ ‘iv,’ and more. We incorporated the auxiliary verbs of the verb “biti” (Engl.: to be) in different forms, such as “sam,” “si,” “je,” “smo,” “ste,” and “su.” Furthermore, we added future tense forms of the verb “hoću” (to want), including “ću,” “ćeš,” “će,” “čemo,” “ćete,”’ and “će.” Lastly, we included numerical expressions like “1/4” (četvrtina), “1/3” (trećina), “1/100” (stotina), and more.

The Bosnian stopwords list consists of 395 entries, while the Croatian list contains 338 en-tries. The reason for the larger number in the Bosnian list is the inclusion of the additional elements mentioned above, specific to the Bosnian language. These additions reflect the linguistic characteristics and specificity's of the Bosnian language.

Overall, the experimental design and materials used in this study ensure the reliability and effectiveness of the developed sentiment lexicon, AnAwords list, and stopwords list for sentiment analysis in the Bosnian language.

## Ethics Statement

The authors have read and follow the ethical requirements for publication in Data in Brief and confirming that the current work does not involve human subjects, animal experiments, or any data collected from social media platforms.

## CRediT authorship contribution statement

**Sead Jahić:** Conceptualization, Software, Investigation, Writing – review & editing, Data curation. **Jernej Vičič:** Conceptualization, Investigation, Writing – review & editing, Data curation, Supervision.

## Data Availability

Sentiment polarity lexicon of Bosnian language (Original data) (Zenodo). Lists of stopwords, polarity shifters and AnAwords of Bosnian language (Original data) (Zenodo).

## References

[1] Jahić S., Vičič J. (2023). Impact of negation and AnAwords on overall sentiment value of the text written in the Bosnian language. Appl. Sci..

[2] T. Cušić, D1.36: report on the Bosnian language, https://european-language-equality.eu/wp-content/uploads/2022/03/ELE___Deliverable_D1_36__Language_Report_Bosnian_.pdf.

[3] BHAS, Cenzus of population, households and dwellings in Bosnia and Herzegovina, 2013 final results, https://dataspace.princeton.edu/handle/88435/dsp0176537424z, 2013.

[4] B. Patra, S. Mazumdar, D. Das, P. Rosso, S. Bandyopadhyay, A Multilevel Approach to Sentiment Analysis of Figurative Language in Twitter, 2018, pp. 281–291. 10.1007/978-3-319-75487-1_22.

[5] Pintarić A., Frleta Z. (2014). Upwards intensifiers in the English, German and Croatian language. Journal for Foreign Languages.

[6] Osmankadić M. (2003). A Contribution to the Classification of Intensifiers in English and Bosnian. Institut za Jezik.

[7] Esuli A., Sebastiani F. (2006). Proceedings of the Fifth International Conference on Language Resources and Evaluation (LREC’06).

[8] F.Årup Nielsen, A new anew: evaluation of a word list for sentiment analysis in microblogs, 2011. arXiv:1103.2903.

[9] P. Stone, D. Dunphy, M. Smith, D. Ogilvie, The General Inquirer: A Computer Approach to Content Analysis, volume 4, 1966. 10.2307/1161774.

[10] Xu L., Zhang C., Li Y., Li J. (2010). Hownet-based Chinese sentiment classification. Expert Syst. Appl..

[11] Kapukaranov B., Nakov P. (2015). Proceedings of the International Conference Recent Advances in Natural Language Processing.

[12] Glavaš G., Snajder J., Dalbelo B. (2012). Proceedings of the International Conference on Text, Speech and Dialogue.

[13] Veselovska K. (2013). Proceedings of the 7th International Conference Slovko, Bratislava.

[14] Jovanoski D., Pachovski V., Nakov P. (2015). Proceedings of the International Conference Recent Advances in Natural Language Processing.

[15] Wawer A. (2012). Extracting emotive patterns for languages with rich morphology. Int. J. Comput. Linguist. Appl..

[16] Okruhlica A. (2013).

[17] K. Kadunc, Določanje sentimenta slovenskim spletnim komentarjem s pomočjo strojnega ucenja, 2016. URL: https://repozitorij.uni-lj.si/IzpisGradiva.php?lang=eng&id=91182.

[18] S. Jahić, J. Vičič, Sentiment polarity lexicon of Bosnian language, 2023. 10.5281/zenodo.7520809.

[19] S. Jahić, J. Vičič, The lists of AnAwords and stopwords are publicly available on the Zenodo repository with 10.5281/zenodo.8021150.

